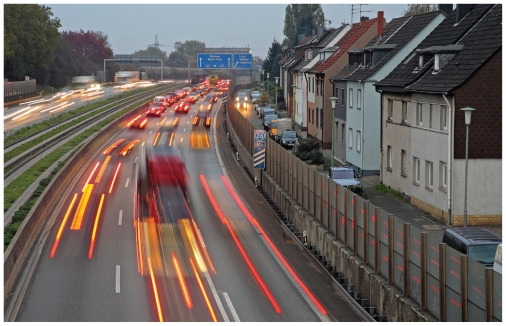# Traffic Trouble: Study Links Diabetes to Vehicular Pollution

**DOI:** 10.1289/ehp.118-a399b

**Published:** 2010-09

**Authors:** Tanya Tillett

**Affiliations:** **Tanya Tillett**, MA, of Durham, NC, is a staff writer/editor for *EHP*. She has been on the *EHP* staff since 2000 and has represented the journal at national and international conferences

There is a well-documented relationship between exposure to particulate matter (PM) in ambient air pollution and risk of developing cardiovascular disease. Subclinical or low-grade inflammation, believed to serve as an intermediary between air pollution and cardiovascular/metabolic health risks, is associated with impaired glucose metabolism, but few studies to date have examined the relationship between air pollution and diabetes. For the first time, a prospective study provides evidence linking exposure to traffic-related air pollution with an increase in the risk of developing type 2 diabetes in women **[*EHP*** **118(9):1273–1279; Krämer et al.]**.

In the current study, researchers investigated the relationship between air pollution exposure and new-onset incident type 2 diabetes using information from the prospective Study on the Influence of Air Pollution on Lung, Inflammation, and Aging (SALIA). The authors also assessed whether baseline inflammation was associated with pollution exposure.

The SALIA cohort is composed of 1,775 women aged 54–55 years without diabetes at enrollment. The women lived in the highly industrialized Ruhr district of Germany or in rural, nonindustrial towns nearby. Using data obtained from cross-sectional surveys administered in 1985–1994 and a follow-up interview in 2006, the investigators analyzed the incidence of type 2 diabetes over 1990–2006. They also collected information on symptoms and diagnoses of respiratory disease, home and occupational exposure to air pollution, smoking status, and socioeconomic status. They took initial height and weight measurements, and collected nonfasting blood serum samples to measure complement factor C3c, a blood protein that served as a marker for subclinical inflammation. They estimated exposure to nitrogen dioxide (NO_2_) and PM, the major components of traffic emissions, by applying land-use regression models.

Between 1990 and 2006, 187 participants (10.5%) were diagnosed with type 2 diabetes. Exposure to traffic-related air pollution and higher levels of C3c in the blood at baseline were both associated with increased diabetes risk. Living within 100 m of a busy roadway was associated with more than double the risk of diabetes for women with a lower education level compared with women in the same group who did not live near a busy roadway; women with higher education who lived near busy roads had no altered risk.

Overall, the researchers observed significant associations with PM and NO_2_ exposure. The slightly stronger associations of risk with NO_2_ exposure than with PM exposure further support a link between traffic-related air pollution exposure and diabetes, since most sources of NO_2_ are traffic-related.

## Figures and Tables

**Figure:**